# Collaboration for Healthy Ageing Through Preventive Home Visits: Identifying Home Visitors' Collaboration Partners

**DOI:** 10.1111/scs.70325

**Published:** 2026-10-03

**Authors:** Anna Nivestam, Albert Westergren, Maria Haak, Gita Hedin

**Affiliations:** ^1^ Department of Nursing and Health Sciences, Faculty of Health Sciences Kristianstad University Kristianstad Sweden; ^2^ Department for Research, Development and Education, Management for Psychiatry and Habilitation Region Skåne Lund Sweden; ^3^ Department of Public Health, Faculty of Health Sciences Kristianstad University Kristianstad Sweden

**Keywords:** community care, health service, healthy ageing, intersectional collaboration, older adults, preventive home visit

## Abstract

**Background and Aim:**

Intersectoral collaboration is essential for promoting healthy ageing. Preventive home visits provide an opportunity for home visitors to support healthy ageing among older adults. However, little is known about which collaboration partners the home visitors engage with. This study aimed to identify collaboration partners engaged by home visitors to achieve positive outcomes from preventive home visits.

**Method:**

This study was based on a re‐analysis of data from five focus group discussions with a total of 16 professionals involved in preventive home visits across five Swedish municipalities. The data was analysed using conventional content analysis. Ethical approval was obtained from the Swedish ethical review authority (reference number 2022‐06054‐01).

**Results:**

The analysis revealed that collaboration with influential entities, such as politicians and the media, was seen as critical for advancing healthy ageing initiatives. Other important partners included those involved in meaningful leisure activities, enabling participation, lifelong learning, and the health‐ and social care sectors.

**Conclusions:**

Home visitors collaborate with a broad range of sectors to promote healthy ageing. Support from leaders in key positions may enhance the effectiveness of these collaborations. The identified partners should be considered in the planning of preventive home visits within a whole‐of‐society approach to ageing.

## 1 Background

Intersectoral collaboration is important for improving healthy ageing [1]. The World Health Organisation has dedicated this decade to healthy ageing, which it defines as “the process of developing and maintaining the functional ability that enables well‐being in older age” (1. p. 28).

Intersectoral collaboration refers to coordinated actions across various societal actors, including governmental, non‐governmental, academic, and older adults' organisations [1]. Additinally, collaboration can be defined as the process by which two or more individuals or entities work together to achieve shared goals or objectives [2].

Although personnel working with older adults' health almost exclusively focus on the micro level, that is, the individual older person [3, 4], they also have the potential to engage across sectors, referred to here as the horizontal engagement, with managers, policymakers, and politicians [5]. Such collaboration beyond the health and welfare sectors is crucial for promoting healthy ageing in a broader societal context. This engagement aims to foster intersectoral collaboration that promotes health by collaborating with various actors outside the health and welfare sectors [5], such as religious organisations, community centres, the transport sector, and housing industries. This horizontal collaboration enhances a holistic approach to public health and emphasises that public health interventions should target intersectoral collaborations across various sectors to succeed [5]. However, little is known about whom personnel working in public health interventions collaborate with to promote healthy ageing.

One well‐known public health intervention for older adults is the preventive home visit [6]. Preventive home visits aim to maintain or improve older adults' health and well‐being by engaging in a health‐promotive dialogue focusing on factors such as social participation, physical health, and mental health [7]. Complex public health interventions such as preventive home visits have been shown to be effective in promoting independence and supporting older adults to live longer in their ordinary housing [8]. Recently, a review also showed that preventive home visits can help older adults maintain functional ability, reduce the need for nursing home admission, and increase the number of visits to primary health care [9].

Extensive systematic reviews and meta‐analyses have shown that preventive home visits can improve health outcomes, including reduced mortality, better physical and mental health, and enhanced quality of life [10, 11, 12]. Swedish studies confirm similar effects [13, 14]. Economic benefits such as fewer care home admissions and reduced healthcare use have also been reported [10, 14]. Recent research highlights further improvements in quality of life, mental health, and social support [9, 15]. However, the positive effects of preventive home visits largely depend on what takes place during the visit and, crucially, on the actions taken afterward. This underscores the importance of having the capacity to advise or refer older adults to a broad range of services and activities that align with their individual needs and preferences.

Usually, older adults without help from the municipal health and welfare system are offered a visit. The age range can vary from around 75 to 85 years [9], and the profession of the person who conducts the visit, the home visitor, can vary, but usually, it is conducted by a nurse [6], and sometimes multiple visits are made by different health personnel [16]. During the visit, the home visitor could advise or refer the older adult to a wide range of other services and activities that fit the older adult's needs and preferences [17]. Therefore, a broad range of collaborative partners is essential to provide appropriate advice. However, the scientific literature about preventive home visits seldom describes what type of collaborators home visitors have beyond the closest team, which might be health personnel working in the same department. In Denmark, research on preventive home visits has a long history [18] and the visits are regulated by law. A recent evaluation of preventive home visits in Denmark showed the variety of collaborators the health personnel had [19]. The personnel in Denmark engaged in communication and collaboration, both internally and externally. They participated in meetings with, for example, the civil society, health care centres, and sports groups. To clarify which collaborators are involved in promoting healthy ageing in connection with preventive home visits, more research is needed. Therefore, this study aims to identify collaboration partners engaged by home visitors to achieve positive outcomes from preventive home visits.

## 2 Method

### 2.1 Design

In the present study, we have used a qualitative design with focus groups. A qualitative research approach was employed, utilising digital focus group discussions as the primary data collection method. Focus groups [20] are designed to foster dynamic interaction among participants, enabling them to share and reflect upon each other's perspectives. This interactive setting encourages the exchange of ideas, leading to a deeper and more comprehensive understanding of the subject matter. Through collaborative dialogue, participants build upon each other's contributions, generating nuanced insights [21]. This method is particularly effective in exploring complex topics, as it captures the collective reasoning and diverse viewpoints of the group [22]. The data was analysed through content analysis. The reporting of the study was guided by the Consolidated Criteria for Reporting Qualitative Research (COREQ) [23].

### 2.2 Data Collection and Procedure

Home visitors were identified through municipal websites and recruited through emails and telephone calls from the first author. The study included persons from all over Sweden who regularly conducted preventive home visits or similar visits. Both oral and written information were given about the study before the focus group discussions. Those who showed interest in participating were invited to an online focus group discussion. Sixteen home visitors from 15 municipalities participated, separated into five discussion groups. In total, two men and 14 women were included, with a median age of 45.5 years (range 31–67), covering the different professions: social workers, assistant nurses, nurses, and public health workers (Table 1). The focus group discussions were conducted by the first and fourth authors. The discussion took place online via a communication platform in October 2023 and lasted between 70 and 100 min. First, the moderator conducted small talk with the home visitors and after that, the discussion questions were asked: Which actors in society do you think could benefit from the information collected? How does the information reach different social actors? Probing questions were: Can you tell me a little more? Can you develop your thoughts?

**TABLE 1:** Demographics of the participants.

	*n* = 16
Gender, *n*	
Female	14
Male	2
Age, years, median (range)	45.5 (31–67)
Education, *n*	
Social worker	3
Assistant nurse	3
Registered nurse	3
Public health	1
Behavioural science/Human resourses	5
Health and occupational rehabilitation	1

### 2.3 Analysis

The transcribed material was analysed according to Hsieh and Shannon's [24] conventional content analysis, with a focus on the aim of identifying the collaborators that home visitors engage with through preventive home visits to promote healthy ageing. First, the material was read several times by the first author to get a comprehensive understanding of the data. Thereafter, the first and fourth authors independently coded one focus group discussion by highlighting collaborators that the home visitors spoke about during the discussions. The authors then compared their initial coding and discussed and agreed on the codes. The first author then continued to code the rest of the material. Examples of codes were “the health care centre”, “the physiotherapist”, “the church”, and “the tennis club”. In the next phase, the first and fourth authors met and discussed the codes to find common meanings among the codes that could form categories. The codes were then grouped into categories. The second and third authors then reviewed the codes and categories that had been created. To enhance the trustworthiness of the results, a summary of the findings was sent by email to four participants, each from a different focus group. All four confirmed that the interpretations accurately reflected their views and experiences. Finally, all authors discussed and agreed on the results.

### 2.4 Ethical Considerations

This study followed the principles of the Declaration of Helsinki throughout the research process. Ethical approval was obtained from the Swedish Ethical Review Authority (reference number 2022‐06054‐01).

Before participating in the focus group discussions, all participants received both written and oral information about the study, and written informed consent was obtained. To protect participant confidentiality, all data were anonymized during transcription, and audio recordings and transcripts were securely stored. In addition to formal procedures, ethical considerations were made to ensure respectful and transparent interactions. The moderators were experienced in qualitative research and took care to foster a respectful and non‐judgmental atmosphere. Their professional roles did not entail supervisory or evaluative authority over participants, which helped reduce power imbalances. At the start of each session, participants were reminded of confidentiality and their right to withdraw at any time without consequence. These considerations helped create a safe space for participants to share their experiences.

## 3 Results

The analysis resulted in a description of collaboration partners engaged by home visitors to achieve positive outcomes from the preventive home visits, presented in five categories: *Collaborators in influential entities; Collaborators for a meaningful leisure time; Collaborators to enable participation; Collaborators for lifelong learning; and Collaborators for good health‐ and social care*.

Overall, the home visitors highlighted many collaborators who seem to be important for promoting healthy ageing, and the prerequisites for finding collaborators seem to vary among the home visitors. It appears important to have time for collaboration and to find new collaborators. Home visitors who are engaged in other activities, besides the preventive home visits, such as organising meeting places for older adults, seem effective in creating new contacts. These contacts could be used during preventive home visits. Sometimes it is explicitly stated in the home visitor's job description that they should communicate with politicians, and the home visitors sometimes see it as a natural part of their job.

### 3.1 Collaborators in Influential Entities

*Collaborators in influential entities* play a central role in decision‐making that promotes healthy ageing. The home visitors said that having a dialogue with collaborators in influential entities such as politicians within the municipality is important to decide on actions that best suit older adults' needs. The analysis revealed that some home visitors had frequent contact with political actors, while others had less interaction with them.

And then I also…present at…our social care committee, municipal council, and such, and I usually take the opportunity to share what I hear (at the home visit) (Focus Group 4).

…well, I'm thinking about the “Senior handyman service”; we have a collaboration between the care administration—I work in the care administration—and the technical administration, where maintenance services and such are located. We have this collaboration, so the connection is quite close. Otherwise, I don't go around lobbying [talking to politicians]; instead, it has to go through formal channels, which means through the immediate manager into the management group and then up to, well, the municipal level, so to speak. (Focus Group 4).

The media is another influential entity that home visitors collaborate with to both communicate about what they are doing related to home visits, as well as inform or advocate about issues related to older adults. The home visitors usually made contact with the media, often traditional media like newspapers.

### 3.2 Collaborators for a Meaningful Leisure Time

The home visitors in our study highlighted several collaborators who could contribute to meaningful leisure time for older adults. These actors were primarily related to physical and social activities. Examples of collaborators mentioned by the home visitors included the ball club, the tennis club, the gym, sports clubs, gymnastics clubs, the Swedish Outdoor Association, the meeting point weaving group, the church, meeting places, daily activities, senior gatherings, and the National Pensioners' Organisation. Usually, the home visitor informed and, in some cases, invited the older adult to specific activities that they could attend. Then it was up to the older adults to contact the organisers of these activities.

Yes, then we have a ball club…which has just started walking football, so they (older adults) will also be invited. (Focus Group 1).

### 3.3 Collaborators to Enable Participation

The home visitors described various actors that can impact older people's opportunities for participation in society, for example to participate in leisure activities. To enable participation, the home visitors tried to find collaborations within the transportation sector. Sometimes, during the visit, the older adult shared that he or she had difficulties with transportation to the activity. One example that the home visitors gave was to help older adults apply for paratransit services.

For services like paratransit, parking permits, and medical transportation, these are recurring topics that I both inform about and assist with, and I usually bring applications and such. (Focus Group 3).

Two home visitors discussed how they tried to collaborate with the transportation sector to arrange a bus to pick up older adults for specific activities. A sense of security is essential for enabling participation, and the police were identified by the home visitors as another important collaborator in promoting security. They invited older adults to meet and engage in discussions with local police officers to help foster this sense of security. Yet other important collaborators that the home visitors identified were for example the Swedish Enforcement Authority, and the banks to promote financial stability. Financial resources were highlighted by the home visitors as a crucial factor enabling older adults to participate, and thus such collaboration was essential. The home visitors said that they had contact with both persons from the Swedish Enforcement Authority, as well as debt counsellors who could come to the older person's home and discuss financial issues.

### 3.4 Collaborators for Lifelong Learning

Another key category of collaborators involved individuals or actors that could promote lifelong learning. This category included young people, the labour market, education, and voluntary organisations for older adults. Collaborators within the voluntary sector, such as the Red Cross, and schools serving children and young people, were often mentioned by the home visitors. The labour market in the form of veteran organisations was also cited as a resource to which home visitors could refer older adults.

The home visitors indicated that they sometimes reached out, or could reach out, to young people who could support older persons in various ways. Thus, the home visitors suggested that they could engage with schools so that older adults could support children, while young people could, in turn, support the older adult.

We have something that is quite beneficial, which becomes a bit relaxed, as turns out when they actually go. And that is lunch for retirees at schools.

It's subsidized, and they go and eat, and it's something that both the children, the youth, and the older adults enjoy when they actually go, but you have to push a little. I've heard… yes, I've pretty much only gotten positive (feedback) when they did go. (Focus Group 4).

These findings demonstrate that collaboration with actors in education, the labour market, and the voluntary sector can support lifelong learning and social inclusion among older adults. Such partnerships may also contribute to meaningful roles and sustained engagement in society, key components of healthy ageing.

### 3.5 Collaborators for Good Health‐ and Social Care

To promote healthy ageing, the home visitors indicated that collaborators in health‐ and social care were important. The home visitors highlighted that, for them to have a contact person at a primary care centre could facilitate collaboration. For example, they said it would be beneficial to have a direct telephone number to the primary care centre. The home visitor could encourage the older person to seek help on their own, or in cases where necessary, assist them with reaching out to the appropriate contact. Additionally, the home visitors suggested that collaborating with hospitals could be beneficial, such as by conducting health‐promoting conversations with patients after discharge.

Although it is called preventive home visits, there are some such questions (related to ill‐health), general ones, like… I have a collaboration with the health centres, so I can recognize vulnerable individuals and refer them onward (Focus Group 2).

Furthermore, the home visitors mentioned having numerous collaborators within the municipal health‐ and social care system, such as physiotherapists, occupational therapists, dementia coordinators, and family support providers. The home visitors made contact with the occupational therapists if the older adult needed assistance devices, or with the dementia coordinator if an older adult wanted guidance about cognitive issues. Most of the home visits had established contact paths with these professions within the municipalities.

## 4 Discussion

Collaboration across various societal actors, including governmental and non‐governmental organisations, academia, and older adults' associations, is crucial for promoting healthy ageing [1]. This study aimed to identify collaboration partners engaged by home visitors within the context of the public health intervention, preventive home visits to older adults. The results highlight that collaborators in influential entities such as politicians and media were seen as integral and significant to the ambition to enhance healthy ageing. Other essential collaborators identified include those related to meaningful leisure activities, actors influencing older adults' participation, lifelong learning facilitators, and persons within the health‐ and social care sectors.

To promote healthy ageing during preventive home visits, it is important to have a wide range of collaboration partners across different organisations. The results showed that home visitors collaborate in *influential entities* (e.g., politicians) as well as *Collaborators for good health‐ and social care* (e.g., healthcare workers). However, formal communication methods may not always exist, and sometimes the communication with collaborators might depend on the home visitors' own network, motivation, interests and engagement. Therefore, establishing agreed‐upon and formal communication channels could facilitate the promotion of healthy ageing. For example, home visitors could maintain regular contact with primary care centres and pensioners' organisations. Forums where these organisations can discuss common issues related to healthy ageing might also facilitate individual contact in specific cases, such as when older adults need help with healthcare issues or finding social activities that match their preferences.

One aspect related to lifelong learning that was seen in the current results was that home visitors collaborate with schools and universities. Strengthening these contacts could lead to more activities related to lifelong learning as well as intergenerational learning. Lifelong learning is an important aspect of healthy ageing described in the scientific literature [25], and meetings between generations can create mutual learning opportunities [26]. Through the experiences of older adults, young people can learn about and reflect on issues such as ageing, what ageing means, and what happens throughout life. Additionally, the older generation is given the opportunity to learn from the younger generations. Consequently, preventive home visits can create opportunities for lifelong learning and intergenerational connections if home visitors continuously collaborate with actors within the educational sector.

The present results demonstrate that collaboration with partners occurs at many levels in society and with entities outside their own organisations. One theoretical perspective commonly used in the literature is the whole‐of‐society approach [27]. This exemplifies a practical application of a whole‐of‐society approach, encompassing more than just health‐ and social care services to promote healthy ageing. Correspondingly, previous research highlights a whole‐of‐society approach as a crucial aspect of healthy ageing [28]. However, it might be possible to engage with more collaborators than those mentioned in the current results. It is important to consider the diversity among home visitors—some visitors might have many collaborators, while others have fewer, depending on their profession and usual contacts. Thus, research like the present study can highlight key collaborators and encourage personnel, such as physiotherapists and occupational therapists, to connect beyond their team for healthy ageing. Previous research highlights, for example, the importance of housing in creating age‐friendly environments that foster healthy ageing [29], and that relational factors are crucial for successful collaboration [30]. Therefore, to expand the range of collaborators that home visitors engage with, it could be beneficial to examine the eight domains of age‐friendly environments [31] and identify additional partners who can contribute to healthy ageing.

While the present study highlights the variety of collaboration partners, for example those related to meaningful leisure time and those who can enable participation, it also raises questions regarding the distribution of responsibility for initiating and maintaining such collaborations in which home visitors are engaged. The findings suggest that much of the collaboration relies on the individual initiative, network, and engagement of the home visitors. However, to fully realise the potential of preventive home visits as a health‐promoting intervention, responsibility should not rest solely on individual professionals. Strategic documents such as the WHO's framework on age‐friendly environments and health promotion models highlight that promoting older adults' health is a shared responsibility across all societal sectors [31]. Furthermore, Swedish municipalities have a legal responsibility to work preventively and promote public health under the Social Services Act [32]. Therefore, municipal authorities and healthcare leaders must take active roles in supporting and coordinating collaborative structures, ensuring that home visitors are not operating in isolation but within a system designed to support intersectoral collaboration for healthy ageing.

### 4.1 Methodological Considerations

Given the limited number of participants in the study, there may be other collaborators not highlighted in the current results. However, in the sample, the representation of home visitors from across Sweden strengthens the results and offers a variety of perspectives. The diverse professions represented among the participants also acknowledge different views on healthy ageing, avoiding the limitation to a single profession that might have fewer collaborators than others. One key limitation of the study is that it is based on a re‐analysis of focus group data originally collected for a different aim. As a result, the interview guide was not specifically tailored to explore the breadth and depth of collaboration partners. This may have influenced the richness and completeness of the data, and some types of collaborations might not have been captured. Moreover, re‐using existing data limited our ability to ask follow‐up questions or explore participants' reflections in greater depth. Despite these limitations, the data were considered sufficiently rich for a meaningful analysis related to the current aim, and the re‐analysis contributes to knowledge development in the field by making use of existing qualitative material. Anyhow, by repeated reading and listening to the material, we found the focus group discussions were rich, and we were able to address the current study aim.

Reusing existing data restricts the opportunity to pose new questions, thereby limiting the ability to acquire more precise and in‐depth insights pertinent to the current study's objectives. For instance, additional information elucidating the mechanisms and rationales behind various collaborations would have been valuable.

## 5 Conclusions

To promote healthy ageing, home visitors conducting preventive home visits to older adults need to collaborate with decision‐makers from various sectors. This study highlights different collaborators working in the health and welfare sector, as well as those involved in lifelong learning, participation, and creating meaningful leisure activities for older adults. These findings are important to consider when designing preventive home visits aimed at promoting healthy ageing, thus adopting a whole‐of‐society approach. The results may have practical implications and serve as inspiration to recognise the diverse range of collaborators needed to promote healthy ageing. It is crucial to be aware of which partners, in the local context, home visitors should collaborate with and how this collaboration can be facilitated.

Future research is needed to explore how organisational structures, policy frameworks, and leadership strategies can support and sustain intersectoral collaboration, as well as to investigate how such collaboration can be systematically integrated into preventive home visits across different municipalities.

## Author Contributions

Conceptualization: A.N., M.H., A.W., and G.H. Methodology: A.N., M.H., A.W., and G.H. Formal analysis: A.N., G.H. Investigation: A.N., G.H. Validation: M.H., A.W. Writing – original draft preparation: A.N. Writing – review and editing: A.N., M.H., A.W., and G.H. Visualisation: A.N., M.H., A.W., and G.H. Project administration: A.N. All authors have read and agreed to the published version of the manuscript.

## Funding

The first author received funding from the Research Platform for Collaboration for Health, Kristianstad University for conducting the focus group discussions. The second, third, and fourth authors conducted the research as part of their employment at Kristianstad University. Moreover, the first author received a postdoc grant from *SSSH Föreningen Södra Sveriges Sjuksköterskehem*, and proofreading of the manuscript was financed by the research group “Patient Reported Outcomes–Clinical Assessment Research and Education (PRO‐CARE)” at Kristianstad University.

## Ethics Statement

This study has followed The Declaration of Helsinki, and ethical approval was obtained from the Swedish ethical review authority (reference number 2022‐06054‐01). Before the focus group, written and oral informed consent was obtained.

## Conflicts of Interest

The authors declare no conflicts of interest.

## Data Availability

The datasets generated during and analysed during the current study are not publicly available due to confidentiality but are available from the corresponding author on reasonable request.

## References

[1] World Health Organization , World Report on Ageing and Health (World Health Organization, 2015).

[2] X. Castañer and N. Oliveira , “Collaboration, Coordination, and Cooperation Among Organizations: Establishing the Distinctive Meanings of These Terms Through a Systematic Literature Review,” Journal of Management 46, no. 6 (2020): 965–1001.

[3] Z. Ebrahimi , H. Patel , H. Wijk , I. Ekman , and P. Olaya‐Contreras , “A Systematic Review on Implementation of Person‐Centered Care Interventions for Older People in Out‐Of‐Hospital Settings,” Geriatric Nursing (New York, NY) 42, no. 1 (2021): 213–224. 10.1016/j.gerinurse.2020.08.004 32863037

[4] T. McCance and B. McCormack , “The Person‐Centred Practice Framework,” in Fundamentals of Person‐Centred Healthcare Practice, 1st ed., ed. B. McCormack , T. McCance , C. Bulley , D. Brown , A. McMillan , and S. Martin (Wiley Blackwell, 2021).

[5] P. P. Valentijn , S. M. Schepman , W. Opheij , and M. A. Bruijnzeels , “Understanding Integrated Care: A Comprehensive Conceptual Framework Based on the Integrative Functions of Primary Care,” International Journal of Integrated Care 13 (2013): e010. 10.5334/ijic.886 PMC3653278 23687482

[6] A. Tourigny , A. Bédard , D. Laurin , et al., “Preventive Home Visits for Older People: A Systematic Review,” Canadian Journal on Aging/La Revue Canadienne du Vieillissement 34, no. 4 (2015): 506–523. 10.1017/S0714980815000446 26526379

[7] M. Markle‐Reid , G. Browne , R. Weir , A. Gafni , J. Roberts , and S. R. Henderson , “The Effectiveness and Efficiency of Home‐Based Nursing Health Promotion for Older People: A Review of the Literature,” Medical Care Research and Review: MCRR 63, no. 5 (2006): 531–569. 10.1177/1077558706290941 16954307

[8] T. F. Crocker , J. Ensor , N. Lam , et al., “Community‐Based Complex Interventions to Sustain Independence in Older People: Systematic Review and Network Meta‐Analysis,” BMJ 384 (2024): e077764. 10.1136/bmj-2023-077764 PMC10955723 38514079

[9] D. B. Ramli , S. Shahar , S. Mat , N. Ibrahim , and N. M. Tohit , “The Effectiveness of Preventive Home Visits on Resilience and Health‐Related Outcomes Among Community Dwelling Older Adults: A Systematic Review,” PLoS One 19, no. 7 (2024): e0306188. 10.1371/journal.pone.0306188 PMC11216600 38950029

[10] R. Elkan , D. Kendrick , M. Dewey , et al., “Effectiveness of Home Based Support for Older People: Systematic Review and Meta‐Analysis,” BMJ 323 (2001): 719–725. 10.1136/bmj.323.7315.719 PMC56889 11576978

[11] E. Mayo‐Wilson , S. Grant , J. Burton , A. Parson , K. Underhill , and P. Motgomety , “Preventive Home Visits for Mortality, Morbidity, and Institutionalization in Older Adults: A Systematic Review and Meta‐Analysis,” PLoS One 9 (2014): e89257. 10.1371/journal.pone.0089257 PMC3951196 24622676

[12] A. E. Stuck , M. Egger , A. Hammer , C. E. Minder , and J. C. Beck , “Home Visits to Prevent Nursing Home Admission and Functional Decline in Elderly People: Systematic Review and Meta‐Regression Analysis,” JAMA 287 (2002): 1022–1028. 10.1001/jama.287.8.1022 11866651

[13] L. Behm , K. Eklund , K. Wilhelmson , et al., “Health Promotion Can Postpone Frailty: Results From the RCT Elderly Persons in the Risk Zone,” Public Health Nursing 33, no. 4 (2015): 303–315. 10.1111/phn.12240 26568469

[14] K. G. Sahlen , C. Lofgren , B. Mari Hellner , and L. Lindholm , “Preventive Home Visits to Older People Are Cost‐Effective,” Scandinavian Journal of Public Health 36 (2008): 265–271. 10.1177/1403494807086983 18519295

[15] Y. L. Tay , N. S. Abu Bakar , R. Tumiran , et al., “Effects of Home Visits on Quality of Life Among Older Adults: A Systematic Review Protocol,” Systematic Reviews 10, no. 1 (2021): 307. 10.1186/s13643-021-01862-8 PMC8650292 34872589

[16] H. Liimatta , P. Lampela , P. Laitinen‐Parkkonen , and K. H. Pitkala , “Preventive Home Visits to Promote the Health‐Related Quality of Life of Home‐Dwelling Older People: Baseline Findings and Feasibility of a Randomized, Controlled Trial,” European Geriatric Medicine 8, no. 5 (2017): 440–445.

[17] A. Nivestam , M. Haak , and A. Westergren , “Recommendations for Healthy Aging as Documented by Health Professionals: A Summative Content Analysis of Health Records,” Primary Health Care Research & Development 24 (2024): e73. 10.1017/S1463423623000671 PMC10790364 38193504

[18] C. Hendriksen , E. Lund , and E. Strømgård , “Consequences of Assessment and Intervention Among Elderly People: A Three‐Year Randomised Controlled Trial,” British Medical Journal (Clinical Research Ed.) 289, no. 6457 (1984): 1522–1524. 10.1136/bmj.289.6457.1522 PMC1443760 6439298

[19] Sundhedsstyrelsen , “Evaluering af forebyggende hjemmebesøg,” 2023, https://www.sst.dk/‐/media/Udgivelser/2023/Forebyggende‐hjemmebesoeg/Evaluering‐af‐forebyggende‐hjemmebesoeg.ashx?sc_lang=da&hash=4E1718CDC9A44BFE695FC521EDDCBCA2.

[20] R. A. Krueger and M. A. Casey , Focus Groups: A Practical Guide for Applied Research, 5th ed. (Sage Publications, 2015).

[21] J. Kitzinger , “The Methodology of Focus Groups: The Importance of Interaction Between Research Participants,” Sociology of Health & Illness 16, no. 1 (1994): 103–121.

[22] S. Dahlin‐Ivanoff and J. Hultberg , “Understanding the Multiple Realities of Everyday Life: Basic Assumptions in Focus‐Group Methodology,” Scandinavian Journal of Occupational Therapy 13, no. 2 (2006): 125–132. 10.1080/11038120600691082 16856469

[23] A. Tong , P. Sainsbury , and J. Craig , “Consolidated Criteria for Reporting Qualitative Research (COREQ): A 32‐Item Checklist for Interviews and Focus Groups,” International Journal for Quality in Health Care 19, no. 6 (2007): 349–357. 10.1093/intqhc/mzm042 17872937

[24] H.‐F. Hsieh and S. E. Shannon , “Three Approaches to Qualitative Content Analysis,” Qualitative Health Research 15, no. 9 (2005): 1277–1288. 10.1177/1049732305276687 16204405

[25] T. Abud , G. Kounidas , K. R. Martin , M. Werth , K. Cooper , and P. K. Myint , “Determinants of Healthy Ageing: A Systematic Review of Contemporary Literature,” Aging Clinical and Experimental Research 34, no. 6 (2022): 1215–1223. 10.1007/s40520-021-02049-w PMC8821855 35132578

[26] E. Tsiloni , E. Dragioti , M. Gouva , S. P. Vassilopoulos , and M. Mentis , “Psychosocial Effects of Intergenerational Learning on Primary School Children and Older Adults: A Systematic Review,” Gerontology & Geriatrics Education 45, no. 2 (2024): 289–322. 10.1080/02701960.2023.2189248 36966565

[27] F. Ortenzi , R. Marten , N. B. Valentine , A. Kwamie , and K. Rasanathan , “Whole of Government and Whole of Society Approaches: Call for Further Research to Improve Population Health and Health Equity,” BMJ Global Health 7 (2022): e009972, 10.1136/bmjgh-2022-009972. PMC9344990 35906017

[28] H. Okayasu and W. Xu , “Developing and Translating Knowledge for Healthy Ageing,” Lancet Regional Health. Western Pacific 35 (2023): 100751. 10.1016/j.lanwpc.2023.100751 PMC10326681 37424678

[29] S. Wu , Y. Fu , and Z. Yang , “Housing Condition, Health Status, and Age‐Friendly Housing Modification in Europe: The Last Resort?,” Building and Environment 215 (2022): 108956.

[30] V. N. Williams , C. Y. Franco‐Rowe , C. Cignetti Lopez , M. A. Allison , and G. J. Tung , “Structural and Relational Factors for Successful Cross‐Sector Collaboration in Home Visiting: A Multiple Case Study,” BMC Health Services Research 24 (2024): 316, 10.1186/s12913-024-10719-4. PMC10921672 38459509

[31] World Health Organization , Age‐Friendly Environments in Europe: A Handbook of Domains for Policy Action (World Health Organization, 2017).

[32] Sweden , Socialtjänstlag (SFS 2025:400) [Social Services Act] (Ministry of Health and Social Affairs, 2025).

